# Impact of carpal tunnel surgery according to pre-operative abnormality of sensory conduction in median nerve: a longitudinal study

**DOI:** 10.1186/1471-2474-14-241

**Published:** 2013-08-15

**Authors:** David Coggon, Georgia Ntani, E Clare Harris, Cathy Linaker, Richard Van der Star, Cyrus Cooper, Keith T Palmer

**Affiliations:** 1 MRC Lifecourse Epidemiology Unit, University of Southampton, Southampton, UK; 2 Department of Clinical Neurophysiology, Wessex Neurological Centre, Southampton General Hospital, Southampton, UK

**Keywords:** Carpal tunnel syndrome, Neurophysiology, Case definition, Validity, Surgery, Outcome

## Abstract

**Background:**

We have previously proposed that sensory nerve conduction (SNC) in the median nerve should be classed as abnormal when the difference between conduction velocities in the little and index fingers is > 8 m/s. In a prospective longitudinal study, we investigated whether this case definition distinguished patients who were more likely to benefit from surgical treatment.

**Methods:**

We followed up 394 patients (response rate 56%), who were investigated by a neurophysiology service for suspected carpal tunnel syndrome. Information about symptoms, treatment and other possible determinants of outcome was obtained through questionnaires at baseline and after follow-up for a mean of 19.2 months. Analysis focused on 656 hands with numbness, tingling or pain at baseline. Associations of surgical treatment with resolution of symptoms were assessed by Poisson regression, and summarised by prevalence rate ratios (PRRs) and associated 95% confidence intervals (95% CIs).

**Results:**

During follow-up, 154 hands (23%) were treated surgically, and sensory symptoms resolved in 241 hands (37%). In hands with abnormal median SNC, surgery was associated with resolution of numbness, tingling and pain (PRR 1.5, 95% CI 1.0-2.2), and of numbness and tingling specifically (PRR 1.8, 95% CI 1.3-2.6). In contrast, no association was apparent for either outcome when median SNC was classed as normal.

**Conclusions:**

Our definition of abnormal median SNC distinguished a subset of patients who appeared to benefit from surgical treatment. This predictive capacity gives further support to its validity as a diagnostic criterion in epidemiological research.

## Background

Diagnosis of carpal tunnel syndrome (CTS) is usually based on a combination of symptoms, signs and findings from neurophysiological investigations. Demonstration of impaired distal sensory nerve conduction (SNC) in the median as compared with the ulnar or radial nerve is generally regarded as the most sensitive neurophysiological marker for the disorder [1]. However, definition of when median SNC is abnormal has been somewhat arbitrary.

We recently examined the relationship of median SNC to symptoms and signs in a consecutive series of patients, who were being investigated for suspected CTS at a general hospital [2]. Even for the combination of symptoms and signs which was associated with the greatest mean reduction in median SNC, there was overlap of the distribution of SNC velocities with that in “normal” hands which had no symptoms or signs. The clearest discrimination from normal hands was achieved with a cut-point of 8 m/s for the difference between little and index finger SNC velocities, and on this basis, we proposed that with the method of testing employed, a value of > 8 m/s was a reasonable definition for an abnormal difference between SNC velocities in the little and index fingers (this would include hands in which no signal could be detected when the index finger was tested, indicating extreme impairment of conduction).

In the absence of a satisfactory diagnostic gold standard, the validity of a case definition is best judged by its practical utility in discriminating categories of illness which might benefit from different preventive strategies (i.e. with different risk factors), or different clinical management (i.e. with different responses to treatment or prognosis) [3]. Using the same case series, we have shown that our definition of abnormal SNC distinguished patient groups which differed importantly in their exposures to risk factors [4]. For example, those with abnormal SNC had markedly higher body mass index (BMI) than controls, whereas no association with BMI was apparent for the group with normal SNC. We here present findings from a follow-up of the earlier study, in which we examined whether our definition of abnormal SNC distinguished patients with different response to surgical treatment.

## Methods

The selection and recruitment of patients has been described previously [2]. They comprised a consecutive series of men and women aged 20–64 years, who attended the neurophysiology department at Southampton General Hospital during January 2007 to September 2009 for investigation of suspected CTS.

At baseline and before the results of nerve conduction studies were known, those who agreed to take part in the study were asked to complete a self-administered questionnaire. Among other things, this covered sex, age, height, weight, smoking habits, somatising tendency, mental health, diabetes, and the occurrence of symptoms during the past month in each hand (numbness, tingling and pain) and in the neck (pain). Information on height and weight was used to derive body mass index (BMI) in kg/m^2^. Questions about somatising tendency were taken from the Brief Symptom Inventory [5], and patients were classified according to the number of common physical symptoms from a total of five (faintness or dizziness, pains in the heart or chest, nausea or upset stomach, trouble getting breath, and hot or cold spells) that had been at least moderately distressing in the past week. Mental health was assessed using questions from the relevant domain of the Short Form-36 (SF-36) Questionnaire [6], and scores were grouped to approximate thirds of the distribution in the study sample (good, intermediate and poor). Where patients reported numbness or tingling in a hand, they were asked to mark the anatomical distribution on a diagram. From this, the distribution was classified according to whether it included most of the area lying within the sensory distribution of the median nerve (“extensive median”), a smaller part of this area (“limited median”), or only other parts of the hand (“other”) [2].

Also at baseline, and again before the results of neurophysiological tests were known, a research nurse (CL) carried out a standardised physical examination of the hands, which included Tinel’s and Phalen’s tests. Details of the methods used are reported elsewhere [2].

Nerve conduction studies were carried out by a physician or clinical physiologist with a Nicolet machine, according to the normal practice of the department. Among other things, measurements were made of orthodromic SNC from the index and little fingers to the wrist, with surface recordings over the median or ulnar nerves proximal to the distal wrist crease. Median SNC was deemed to be abnormal if there was no detectable signal when the index finger was tested, or if the difference in SNC velocity between the little and the index finger was > 8 m/s.

At the time of completing the baseline questionnaire, participants were asked whether they would be willing to receive a follow-up questionnaire. Those who agreed were sent a further, shorter questionnaire by post after an interval of approximately 18 months (followed by a reminder to those who did not respond). This asked about treatment for hand and arm symptoms since baseline (surgery on the wrist or hand; injections; and physical therapy), and about numbness, tingling and pain in the hands during the past four weeks.

Statistical analysis was carried out with Stata version 11.1 software. As in our earlier report [2], analysis was based on hands, and was restricted to those which had not been treated surgically for CTS before baseline. We also excluded hands in which there had been no numbness, tingling or pain in the four weeks before the baseline questionnaire was answered.

For the hands on which follow-up information was obtained, we first used Poisson regression to explore baseline predictors of subsequent surgery. To account for the hierarchical structure of the data, we employed multi-level modelling, clustering by patient. Associations were summarised by prevalence rate ratios (PRRs) with associated 95% confidence intervals (CIs).

Next, we examined the frequency with which symptoms had resolved by follow-up (i.e. had been absent throughout the four weeks before answering the follow-up questionnaire) according to baseline characteristics and treatments received during the follow-up period. Two parallel sets of multilevel Poisson regression analyses were carried out, the first with resolution of all sensory symptoms (numbness, tingling and pain) as the outcome, and the second with resolution of numbness and tingling as the outcome. The second set of analyses was restricted to hands in which numbness or tingling had been reported at baseline.

Finally, we compared the association of surgical treatment with symptom resolution according to the presence or absence of abnormal median SNC and other clinical features at baseline. The statistical significance of differences in association was quantified by examining the interaction between the baseline feature and surgical treatment in a regression analysis which took resolution of symptoms as the dependent variable.

Ethical approval for the study was provided by the Southampton and South West Hampshire NHS Research Ethics Committee.

## Results

**Figure 1:**
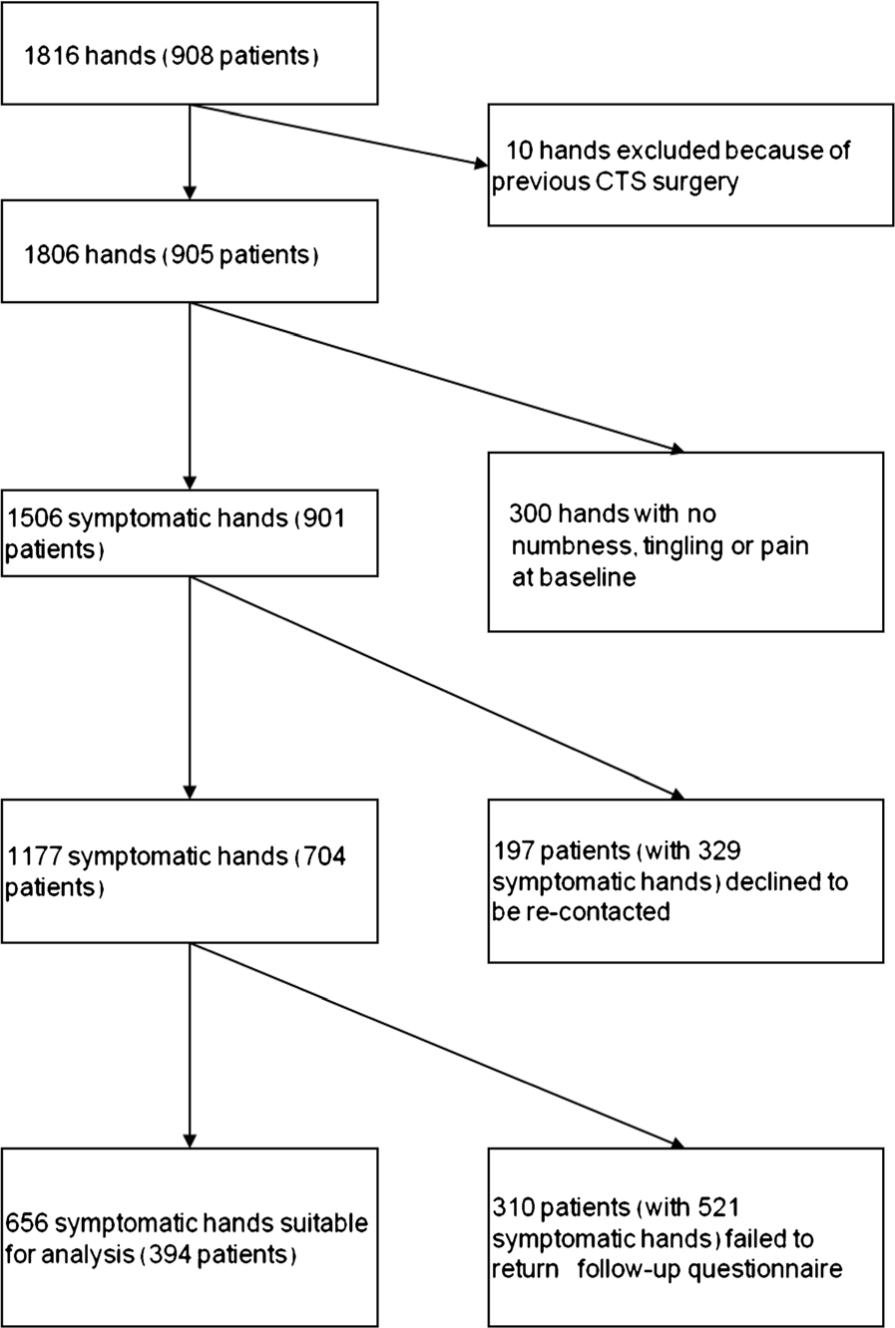
Hands and patients analysed and reasons for exclusions.

After exclusion of patients who declined to take part in the study (27%) and 10 hands that had already been treated surgically for CTS, our previous analysis was based on 1806 hands in 905 patients [2]. They included 1506 hands with numbness, tingling or pain in 901 patients, 704 of whom agreed to receive a follow-up questionnaire (Figure 1). Among these 704 patients, 394 (56%) completed the follow-up questionnaire, giving a total of 656 originally symptomatic hands that could be included in the current analysis (both hands from 262 patients and one hand from 132 patients). Numbness or tingling had been reported in 633 of the hands at baseline, while the other 23 hands had been painful but without numbness or tingling.

Table 1 shows the numbers of hands analysed according to characteristics assessed at baseline. Response rates at follow-up tended to be higher in women than in men, and at older ages. However, there were no important differences in the completeness of follow-up data according to nerve conduction or other clinical findings at baseline. Among the 394 patients who provided follow-up data, the mean interval from baseline to follow-up was 19.2 months (median 18.9, range 15.0 to 31.0, all but four patients between 17 and 24 months).

**Table 1:** Number of hands analysed according to characteristics assessed at baseline

**Baseline characteristic of patient or hand**	**Hands with symptoms at baseline**		**Hands analysed**		
	**All patients**	**Agreed to be followed up**			
	**N**	**N**	**N**	**%**^**a**^	**%**^**b**^
**Sex**					
Male	480	371	180	37.5	48.5
Female	1026	806	476	46.4	59.1
**Age (years)**					
<40	379	269	93	24.5	34.6
40–49	486	382	193	39.7	50.5
50–59	481	395	271	56.3	68.6
≥60	160	131	99	61.9	75.6
**Distribution of numbness/tingling**					
Other	168	142	80	47.6	56.3
Limited median	392	314	172	43.9	54.8
Extensive median	899	677	381	42.4	56.3
None	47	44	23	48.9	52.3
**Tinel’s test**					
Negative	873	674	371	42.5	55.0
Positive	430	324	192	44.7	59.3
Missing	203	179	93	45.8	52.0
**Phalen’s test**					
Negative	488	376	222	45.5	59.0
Positive	819	626	347	42.4	55.4
Missing	199	175	87	43.7	49.7
**Median nerve sensory conduction**					
Normal	747	600	322	43.1	53.7
Abnormal	618	474	267	43.2	56.3
Missing	141	103	67	47.5	65.0

^a^% of all hands with symptoms at baseline.

^b^% of hands with symptoms at baseline in patients who agreed to receive follow-up questionnaire.

In total, 154 hands (23%) had been treated surgically during the follow-up period, 47 (7%) by injection (of which 18 were also treated surgically), and 88 (13%) by physical therapies (of which 31 were also treated surgically). The strongest baseline predictor of subsequent surgery was abnormal median SNC (PRR after adjustment for other risk factors 3.6, 95% CI 2.3-5.5) (Table 2). After allowance for neurophysiological abnormality, surgery was also more common when Phalen’s test was positive (PRR 2.4, 95% CI 1.4-4.0). However, associations with numbness/tingling in an extensive median distribution, and with positive Tinel’s test, both disappeared after adjustment for other predictors.

**Table 2:** Baseline predictors of carpal tunnel surgery

**Risk factor**	**Hands**	**Hands treated by surgery**					
	**N**	**N**	**(%)**	**PRR**^**a**^	**(95% CI)**	**PRR**^**b**^	**(95% CI)**
**Sex**							
Male	180	44	24.4	1		1	
Female	476	110	23.1	0.9	(0.7–1.3)	1.0	(0.7–1.4)
**Age (years)**							
<40	93	23	24.7	1		1	
40–49	193	46	23.8	1.0	(0.6–1.6)	0.8	(0.5–1.4)
50–59	271	61	22.5	0.9	(0.6–1.5)	0.9	(0.6–1.5)
≥60	99	24	24.2	1.0	(0.6–1.7)	0.9	(0.5-1.6)
**Distribution of numbness/tingling**							
Other	80	13	16.3	1		1	
Limited median	172	30	17.4	1.1	(0.6–2.1)	0.9	(0.5–1.8)
Extensive median	381	109	28.6	1.8	(1.0–3.1)	1.2	(0.7–2.1)
None	23	2	8.7	-	-		
**Tinel’s test**							
Negative	371	64	17.3	1		1	
Positive	192	68	35.4	2.1	(1.5–2.9)	1.2	(0.8–1.7)
Missing	93	22	23.7	-		-	
**Phalen’s test**							
Negative	222	20	9.0	1		1	
Positive	347	112	32.3	3.6	(2.2–5.8)	2.4	(1.4–4.0)
Missing	87	22	25.3	-		-	
**Median sensory nerve conduction**							
Normal	322	27	8.4	1		1	
Abnormal	267	101	37.8	4.5	(3.0–6.9)	3.6	(2.3–5.5)
Missing	67	26	38.8	-		-	

^a^Each risk factor analysed independently.

^b^All risk factors analysed in a single regression model.

Overall, 241 hands (37%) were free from pain and sensory disturbance at the time of follow-up, while the occurrence of numbness or tingling had resolved in 312 (49%) of the 633 hands in which these symptoms had been reported at baseline. Table 3 shows the frequency of symptom resolution in relation to baseline characteristics and treatment received during follow-up. Resolution of symptoms was unrelated to sex or age, but was more frequent in patients treated by surgery (PRR 1.4 for both outcomes). After allowance for these variables, it was less likely in current smokers (PRR 0.7 for both outcomes) and in patients with a strong tendency to somatise (PRR 0.7 for both outcomes). In addition, complete resolution of sensory symptoms, including pain as well as numbness and tingling, was less common when neck pain was reported at baseline (PRR 0.7, 95% CI 0.6-0.9) and more common in patients with diabetes (PRR1.6, 95% CI 1.0-2.5), while numbness and tingling were less likely to resolve when BMI was ≥30 kg/m^2^ (PRR 0.7, 95% CI 0.5-1.0).

**Table 3:** Frequency of symptom resolution in hands according to baseline characteristics and treatment during follow-up

**Predictor**	**Pain, numbness or tingling**					**Numbness or tingling**				
	**No. at baseline**	**Symptoms resolved at follow-up**				**No. at baseline**	**Symptoms resolved at follow-up**			
		**N**	**(%)**	**PRR**^**a**^	**(95% CI)**		**N**	**(%)**	**PRR**^**a**^	**(95% CI)**
**Sex**										
Male	180	72	40.0	1		176	90	51.1	1	
Female	476	169	35.5	0.9	(0.7–1.2)	457	222	48.6	0.9	(0.7–1.2)
**Age (years)**										
<40	93	35	37.6	1		91	46	50.5	1	
40–49	193	66	34.2	0.9	(0.6–1.3)	192	85	44.3	0.9	(0.6–1.3)
50–59	271	110	40.6	1.1	(0.7–1.6)	256	144	56.3	1.1	(0.8–1.6)
≥60	99	30	30.3	0.8	(0.5–1.3)	94	37	39.4	0.8	(0.5–1.2)
**Treated by surgery**										
No	502	170	33.9	1		481	216	44.9	1	
Yes	154	71	46.1	1.4	(1.0–1.8)	152	96	63.2	1.4	(1.1–1.8)
**Treated by injection**										
No	609	230	37.8	1		587	293	49.9	1	
Yes	47	11	23.4	0.6	(0.3–1.1)	46	19	41.3	0.8	(0.5–1.2)
**Treated by physical therapy**										
No	568	211	37.1	1		546	275	50.4	1	
Yes	88	30	34.1	0.9	(0.6–1.3)	87	37	42.5	0.8	(0.6–1.2)
**BMI**										
<25	201	72	35.8	1		191	107	56.0	1	
≥25 and <30	228	90	39.5	1.1	(0.8–1.5)	221	109	49.3	0.8	(0.6–1.1)
≥30	211	75	35.5	0.9	(0.7–1.3)	205	90	43.9	0.7	(0.5–1.0)
Missing	16	4	25.0	0.6	(0.2–1.7)	16	6	37.5	0.6	(0.3–1.3)
**Smoking**										
Never	313	122	39.0	1		302	163	54.0	1	
Ex-	213	79	37.1	0.9	(0.7–1.2)	208	101	48.6	0.8	(0.6–1.1)
Current	122	36	29.5	0.7	(0.5–1.0)	115	44	38.3	0.7	(0.5–1.0)
Missing	8	4	50.0	1.3	(0.5–3.5)	8	4	50.0	1	(0.4–2.6)
**Neck pain**										
No	302	132	43.7	1		291	156	53.6	1	
Yes	354	109	30.8	0.7	(0.6–0.9)	342	156	45.6	0.9	(0.7–1.1)
**Number of distressing somatic symptoms**										
0	141	62	44.0	1		138	76	55.1	1	
1	192	75	39.1	0.9	(0.6–1.3)	181	104	57.5	1.1	(0.8–1.4)
≥2	323	104	32.2	0.7	(0.5–1.0)	314	132	42.0	0.7	(0.6–1.0)
**Mental health**										
Good	226	76	33.6	1		218	98	45.0	1	
Intermediate	200	79	39.5	1.2	(0.9–1.6)	193	101	52.3	1.2	(0.9–1.5)
Poor	225	81	36.0	1.1	(0.8–1.5)	217	108	49.8	1.1	(0.9–1.5)
Missing	5	5	100.0	3.2	(1.3–7.9)	5	5	100.0	2.3	(0.9–5.6)
**Diabetes**										
No	616	217	35.2	1		593	288	48.6	1	
Yes	40	24	60.0	1.6	(1.0–2.5)	40	24	60.0	1.1	(0.7–1.8)

^a^Each predictor analysed independently with adjustment for sex, age and surgical treatment.

Table 4 compares associations between symptom resolution and surgical treatment according to clinical features assessed at baseline. Risk estimates are presented with and without adjustment for the five risk factors in Table 3 which showed significant (p < 0.05) associations with resolution of symptoms (smoking habits, somatising tendency, neck pain, diabetes and BMI). The adjustment had only a small impact. Among patients with abnormal median SNC, surgery was associated with resolution both of numbness, tingling and pain (adjusted PRR 1.5, 95% CI 1.0-2.2) and of numbness and tingling specifically (adjusted PRR 1.8, 95% CI 1.3-2.6). In contrast, no association with surgery was apparent for either outcome where median SNC was classed as normal. Similarly, there was evidence of a differential outcome after surgery where numbness and tingling were originally present in regions of the hand falling exclusively within the sensory distribution of the median nerve. However, negative findings from both Tinel’s and Phalen’s tests did not preclude a better outcome in patients treated surgically. The difference in associations between surgery and resolution of numbness and tingling according to median SNC abnormality was of borderline statistical significance (p = 0.05).

**Table 4:** Frequency of symptom resolution in hands treated or not treated by surgery according to clinical features assessed at baseline

**Clinical feature**	**Numbness, tingling or pain**				**Numbness or tingling**			
	**No surgery**	**Surgery**			**No surgery**	**Surgery**		
	**N (%) resolved**	**N (%) resolved**	**PRR**^**a**^	**PRR**^**b**^	**N (%) resolved**	**N (%) resolved**	**PRR**^**a**^	**PRR**^**b**^
			**(95% CI)**	**(95% CI)**			**(95% CI)**	**(95% CI)**
**Distribution of numbness/tingling**								
Other	22 (32.8)	2 (15.4)	0.5 (0.1–2.0)	0.6 (0.1–2.5)	31 (46.3)	6 (46.2)	1.0 (0.4–2.4)	1.2 (0.5–3.0)
Limited median	52 (36.6)	17 (56.7)	1.5 (0.9–2.7)	1.7 (0.9–3.1)	69 (48.6)	20 (66.7)	1.4 (0.8–2.3)	1.5 (0.9–2.6)
Extensive median	89 (32.7)	52 (47.7)	1.5 (1.0–2.1)	1.4 (1.0–2.1)	116 (42.6)	70 (64.2)	1.5 (1.1–2.0)	1.6 (1.1–2.2)
**Tinel’s and Phalen’s tests**								
Both negative	70 (39.1)	12 (63.2)	1.6 (0.9–3.0)	1.5 (0.8–2.7)	82 (50.3)	14 (77.8)	1.5 (0.9–2.7)	1.4 (0.8–2.6)
At least one positive	80 (31.4)	53 (46.5)	1.5 (1.0–2.1)	1.4 (1.0–2.0)	106 (42.1)	74 (65.5)	1.6 (1.2–2.1)	1.6 (1.1–2.1)
Missing	20 (29.4)	6 (28.6)	1.0 (0.4–2.4)	0.9 (0.3–2.5)	28 (42.4)	8 (38.1)	0.9 (0.4–2.0)	0.9 (0.4–2.1)
**Median nerve sensory conduction**								
Normal	93 (31.5)	8 (29.6)	0.9 (0.5–1.9)	0.9 (0.4–1.9)	129 (46.1)	9 (34.6)	0.8 (0.4–1.5)	0.8 (0.4–1.6)
Abnormal	57 (34.3)	51 (50.5)	1.5 (1.0–2.1)	1.5 (1.0–2.2)	67 (41.1)	72 (72.0)	1.8 (1.3–2.4)	1.8 (1.3–2.6)
Missing	20 (48.8)	12 (46.2)	0.9 (0.5–1.9)	1.4 (0.6–3.3)	20 (52.6)	15 (57.7)	1.1 (0.6–2.1)	1.6 (0.7–3.6)
**All hands**	170 (33.9)	71 (46.1)	1.4 (1.0–1.8)	1.3 (1.0–1.7)	216 (44.9)	96 (63.2)	1.4 (1.1–1.8)	1.4 (1.1–1.8)

^a^Unadjusted.

^b^Adjusted for BMI, smoking habits, neck pain, number of distressing somatic symptoms and diabetes at baseline.

## Discussion

In this follow-up investigation, the definition of abnormal median SNC that we had previously proposed distinguished a group of patients which appeared to benefit from surgery from another which did not. This further supports the utility of the definition as a diagnostic criterion in epidemiological research.

In longitudinal studies, the response rate most relevant to internal validity is that among subjects who enter follow-up – in our study, patients who consented at baseline to receive a second questionnaire. The response rate that we were able to achieve (56% overall) was less than ideal, and as in many studies, was lower in men and at younger ages. Nevertheless, important bias would have resulted only if responders were atypical as regards the relation of baseline characteristics to outcomes of interest – principally the resolution of numbness, tingling and pain, and its association with surgery. That response rates varied little according to clinical findings at baseline (Table 1) makes it unlikely that associations between symptom resolution and surgery would be biased differentially in relation to these clinical features.

A greater challenge to interpretation is the possibility of confounding by factors influencing selection for surgery. Although randomised controlled trials attest to the benefit of surgical decompression of the carpal tunnel in CTS [7], the associations which we observed between surgical treatment and symptom resolution may not fully reflect the impact of surgery. It could be, for example, that a decision was taken not to operate on some patients because their symptoms were already improving spontaneously, in which case the benefits of surgery would tend to be underestimated in our analysis. To minimise the potential for such confounding, we adjusted for risk factors at baseline which were identified as carrying an adverse prognosis independent of surgical treatment, and reassuringly, this adjustment had only minor impact on the risk estimates of main interest. We cannot rule out a residual effect of other, unrecognised confounders, but to produce important bias in relation to our study question, such confounding would have to be differential according to baseline nerve conduction results, which seems unlikely. In particular, there is no reason to expect that surgeons would be more likely to operate on patients with a poorer chance of responding to surgery if they had normal as compared with abnormal median nerve conduction.

Another limitation was the modest statistical power to demonstrate differences in associations of symptom resolution with surgery according to baseline characteristics. Thus although surgery was clearly associated with the disappearance of symptoms in hands with abnormal median SNC, and there was no indication of any benefit from surgery in those with normal median SNC, this divergence was only of borderline statistical significance. That said, our investigation was larger than most previously reported studies of outcome following surgery for CTS [8], and had the advantage of referent data from patients who were not treated surgically.

As would be expected, abnormality of median SNC was a strong predictor of surgical treatment (PRR 3.6, 95% CI 2.3-5.5). However, surgery was by no means limited to hands with abnormal median SNC according to our definition. This is not surprising since the definition, which was proposed for use in epidemiological studies and not in clinical practice, was formulated only after the collection of all baseline data for the study was complete. Moreover, it did not take account of other neurophysiological findings – for example, on motor nerve conduction. The importance of other clinical features is evidenced in the higher rate of surgery when Phalen’s test was positive, even after account was taken of whether median SNC was abnormal (Table 2).

After allowance for any surgical treatment, symptom resolution was less frequent in current smokers and in patients who tended to somatise. An adverse prognostic influence of smoking has not been reported previously, but somatising tendency has been found to predict persistence of musculoskeletal pain in other circumstances [9,10], presumably because it increases patients’ awareness of, and causes them to dwell on, symptoms which others would dismiss. Complete resolution of sensory symptoms was also less likely in patients who reported neck pain at baseline, perhaps because in some of these patients, hand symptoms were attributable to pathology in the neck that was less likely to improve over time. In contrast, diabetes was associated with higher resolution of symptoms. In addition, numbness and tingling resolved less frequently in patients with high BMI. Obesity is a known risk factor for CTS [11-16], but has not been clearly associated with worse outcome following surgery [17]. The comparisons that we made of associations between surgery and symptom resolution were all carried out with and without adjustment for these prognostic variables.

Although many studies have examined outcomes after surgery for CTS according to pre-operative neurophysiological findings [18-35], these have not entailed comparisons with patients who had similar neurophysiological results but were not treated surgically. Thus, they do not allow assessment of the extent to which improvements in symptoms could be attributed to surgery, or of whether patients with particular neurophysiological abnormalities benefitted more from surgical treatment. Because of limited statistical power and the possibility of uncontrolled residual confounding, we cannot draw firm conclusions on this second question. However, our results support the hypothesis that abnormal median SNC, as we have defined it, distinguishes hands that are more likely to benefit from surgical decompression of the carpal tunnel. They also suggest that surgery is unlikely to be of value when numbness and tingling do not affect any parts of the hand that lie exclusively within the sensory distribution of the median nerve. In contrast, when both Tinel’s and Phalen’s tests were negative, there was still an apparent benefit from surgical treatment.

## Conclusions

We have already demonstrated that our proposed definition of abnormal median SNC distinguished disease with distinctive risk factors [4]. That it appears also to predict benefit from surgical treatment gives further support to its validity as a diagnostic criterion in epidemiological research.

## Competing interests

The authors declare that they have no competing interests.

## Authors’ contributions

DC and KTP conceived the study and oversaw its conduct. RVdS and CC contributed to the study design. CL and ECH carried out the data collection and helped prepare the data for analysis. GN carried out the statistical analysis. DC wrote the first draft of the manuscript. All of the authors contributed to revision and finalisation of the manuscript. All authors read and approved the final manuscript.

## Pre-publication history

The pre-publication history for this paper can be accessed here:

http://www.biomedcentral.com/1471-2474/14/241/prepub
